# Transnational social networks, health, and care: a systematic narrative literature review

**DOI:** 10.1186/s12939-021-01467-6

**Published:** 2021-06-12

**Authors:** Inez Roosen, Sarah Salway, Hibbah Araba Osei-Kwasi

**Affiliations:** 1 Faculty of Health, Medicine and Life Sciences, Maastricht University, Maastricht, the Netherlands; 2 Department of Sociological Studies, University of Sheffield, Sheffield, UK; 3 Department of Geography, University of Sheffield, Sheffield, UK

**Keywords:** Social remittances, Transnational social exchanges, Health, Wellbeing, Migrants, Remain behind, Transnational networks

## Abstract

While transnational social ties and exchanges are a core concern within migration studies, health researchers have often overlooked their importance. Continuous and circular exchanges of information within transnational networks, also defined as social remittances, facilitate the diffusion of innovations, potentially driving contemporary social and cultural change. Influences on health, wellbeing, and care-seeking are important, but under-researched, dimensions for consideration. We undertook a systematic narrative evidence synthesis to describe the current state of knowledge in this area and to identify gaps and future directions for health researchers to take. Between April 2017 and May 2019, an iterative series of searches in Medline, Embase, PsycINFO and PubMed, plus backward and forward citation searches identified 1173 potential papers. Screening resulted in 36 included papers, eighteen focused on migrant populations and eighteen on those who remain behind. The top three health topics were health-seeking strategies, sexual and reproductive health issues, and healthcare support. And, while not always explicitly identified, mental health and wellbeing was a further prominent, cross-cutting theme. Articles on migrant populations were all conducted in the global North and 13 out of 18 used qualitative methods. Five main themes were identified: therapeutic effect of the continuing social relationships, disrupted social relationships, hybridisation of healthcare, facilitation of connections to healthcare providers, and factors encouraging or undermining transnational social exchanges. Papers concerned with those who remain behind were mainly focused on the global South and used a mix of qualitative and quantitative approaches. Four main themes were identified: transnational transfer of health-related advice, norms, and support; associations between migrant linkages and health behaviours/outcomes; transnational collective transfer of health knowledge; and power and resistance in exchanges. Findings suggest that transnational social exchanges can both support and undermine the health of migrants and those who remain behind. This review confirms that the volume and quality of research in this area must be increased so that health policy and practice can be informed by a better understanding of these important influences on the health of both migrants and those who remain behind.

## Introduction

Humans are social beings, part of social networks. Social selection and social influence shape the attitudes, values, norms, and behaviours of network members, leading to similar behaviours within a group [1, 2]. Even our health, wellbeing, and care-seeking behaviours are influenced by our social networks, both on a personal and group-based level [3].

With the increasing developments in information technologies, we can connect and exchange information even faster than before within our social network [4, 5]. The connection and exchange of information within social networks is especially interesting when considering migrant populations, as they are known to be living in (at least) two worlds – with one foot in their destination and another in their origin [6]. Glick Schiller, Basch, and Blanc-Szanton first raised the term – transnationalism – in 1992, meaning that social networks cross borders connecting migrants with their network members who remain behind in the country of origin. Within these transnational networks an exchange of information occurs. The communication between migrants and members in their home country can be seen as an informal transnational exchange of human and social capital, facilitated by telecommunication technologies [7]. This communication flow, entailing ideas, norms, values, practices, and behaviours – also termed ‘social remittances’, as coined by Peggy Levitt in 1998, can be usefully extended to ‘transnational social exchanges’ – reflecting the way in which these movements happen circularly and continuously. These transnational social exchanges allow for the diffusion of innovations, driving contemporary social and cultural (ex)change [6, 8–10].

The study of migration has, until relatively recently, been dominated by Western Anglo-Saxon scholars interested in the movement of people from the global South to the global North and has frequently been intertwined with policy-making concerns around the integration of migrant populations within Northern destination societies [11]. More recently, this narrow orientation has been challenged, with the importance of understanding migration processes within the global South [12] and the ongoing relationships between diasporic communities and their countries of origin being increasingly recognised [8, 9, 13]. Nevertheless, while transnational social ties have become a core area of enquiry within migration studies, health and care researchers have commonly overlooked the importance of transnational networks and social exchanges in their research [13, 14]. However, understanding transnational networks and the associated social exchanges is relevant to comprehending and meeting health needs of both migrants and those who remain behind. Villa-Torres et al. [13] provided a solid basis in their systematic literature review about the use of a transnational perspective in researching migrant health and its outcomes. They identified several relevant components concerning transnational migrant health, such as migrants’ ability to return temporarily for health care and the importance of active transnational networks to gain access to health-related information or supplies (such as traditional/home remedies). This review [13] did not, however, focus specifically on transnational social exchanges and their influences on health. Furthermore, one of the specific gaps identified by Torres et al. [13] is research on the health of those who remain behind. Therefore, we addressed these outstanding gaps by conducting a systematic narrative literature review, focusing on transnational social exchanges and their role in the health and wellbeing of migrants and those who remain behind. The overarching review question of this study was:

*What role do social remittances (transnational social exchanges) play in shaping health-related practices and health outcomes for*

- *migrants, and*
- *those who remain behind in the country of origin (with personal links to migrants)?*

## Method

We undertook a systematic narrative literature review using the software EPPI-Reviewer, ensuring transparency and standardisation during the process [15].

### Search strategy

The search for this systematic narrative literature review took place in four steps, as described below.

#### Step 1: initial search

This systematic narrative review was based on initial searches conducted in April 2017 in three electronic databases - Medline, Embase, and PsycINFO. We developed and employed a bespoke set of search terms relating to two domains of interest - social exchanges and migrants (please see Table 1 for more information). The initial search strategy did not include health and care search terms as we tried to cover a broad perspective of studies on our topic and used our selected databases (Medline (biomedical), Embase (biomedical and pharmacological), and PsycINFO (interdisciplinary behavioural and social science research)) to provide relevant health and care related articles.

**Table 1:** Database search terms employed in initial searches April 2017

# Search	Search terms used *(searches in Medline, Embase, and PsycInfo)*
DOMAIN 1: SOCIAL EXCHANGES	
1.	Social remittances.mp.
2.	Social remittances.mp. [mp = title, abstract, heading word, drug trade name, original title, device manufacturer, drug manufacturer, device trade name, keyword, floating subheading]
3.	transnational exchange$.tw.
4.	(social adj5 norm$).tw.
5.	(social adj5 practice$).tw.
6.	(social adj5 identit$).tw.
7.	Cultural diffusion.mp. [mp = title, abstract, heading word, drug trade name, original title, device manufacturer, drug manufacturer, device trade name, keyword, floating subheading]
8.	((change$ or alter* or differen$) adj10 normative value$).tw.
9.	Social remitting.mp. [mp = title, abstract, heading word, drug trade name, original title, device manufacturer, drug manufacturer, device trade name, keyword, floating subheading]
10.	social network$.tw.
11.	social exchange$.tw.
12.	kinship network$.tw.
13.	Friendship network$.tw.
14	transnational transfer$.tw.
15.	transnational transmission$.tw.
16.	transnational communit$.tw.
17.	Knowledge transfer.tw.
18.	Information exchange.tw.
19.	(transfer adj5 information).tw.
20.	1 or 2 or 3 or 4 or 5 or 6 or 7 or 8 or 9 or 10 or 11 or 12 or 13 or 14 or 15 or 16 or 17 or 18 or 19
DOMAIN 2: MIGRANTS	
21.	immigrant*.tw.
22.	emigrant$.tw.
23.	Immigration.tw.
24.	Emigration.tw.
25.	Source country.tw.
26.	Origin country.tw.
27.	Destination country.tw.
28.	Host country.tw.
29.	Stay behind.tw.
30.	stayer$.tw.
31.	(mover or movers).tw.
32.	Remain behind.tw.
33.	refugee$.tw.
34.	asylum seeker$.tw.
35.	21 or 22 or 23 or 24 or 25 or 26 or 27 or 28 or 29 or 30 or 31 or 32 or 33 or 34
COMBINING DOMAIN 1 (SOCIAL EXCHANGES) AND DOMAIN 2 (MIGRANTS)	
36.	20 and 35

#### Step 2: supplementary search – focus on those who remain behind

During our initial search, we observed a limited number of studies focusing on those who remain behind. Hence, we decided to perform an additional search for these studies in PubMed. To receive more focused outcomes on our topic we decided using the main keywords [migrant] OR [immigrant] AND [transnational] OR [social exchanges] AND [health] up to January 2018. Only studies published in English and focused on those who remain behind were included. Furthermore, no additional filters were applied.

#### Step 3: general supplementary search

In addition, to expand our studies from the initial search, backward and forward citation searches were applied to all papers included via the database searches up to February 2018.

#### Step 4: final search to update studies

Finally, to update previous searches, we employed a forward citation search up to May 2019 for two seminal papers on social remittances [8, 9].

### Screening and study eligibility criteria

Empirical papers using any qualitative or quantitative methodology that explored topics related to [1] transnational networks/connections, social remittances/transnational social exchanges, *and* [2] health practices or healthcare use or health and wellbeing outcomes were included. All studies focused on transnational migrants (people living outside of their country of birth) or individuals who ‘remain behind’ (those identified as having a migrant family member or significant other). All studies were in English; had no restrictions on date, country of origin or destination, and migrant status.

We undertook a two-stage screening process. In stage one, all papers were screened on title and abstract by one of three researchers to remove any that were obviously out of remit. The first ten papers screened by each reviewer were cross-checked by a second reviewer to ensure consistent application of the inclusion criteria. Any uncertain papers were referred to group discussion and collective agreement on inclusion/exclusion. In the second stage, a full-text screen was applied to the remaining eligible papers. During the full-text screening, consistency checks again involved cross-checking the first ten papers and discussing any unsure papers within the group.

### Quality assessment

For the quality assessment of our included studies, we decided to adopt an approach that captured the dimensions we felt were most important to evaluate our included studies. We created two quality assessment forms, one for quantitative and mixed-methods studies and another for qualitative studies, covering a customised set of dimensions such as research design, data collection, data analysis, and reporting [16].

Each article was assessed, and each component was either considered sufficient or insufficient. A summary assessment was given according to the following criteria: ‘Low’ (< 4 components assessed as sufficient), ‘Moderate’ (4–6 components assessed as sufficient), and ‘Good’ (7–9 components assessed as sufficient). Papers were not completely excluded based on study quality, but rather the contribution of findings from poorer studies to the synthesis was stated. An overview of the quality assessments can be found in Tables 2, 3, 4, and 5.

**Table 2:** Quality assessment – Qualitative tool - Migrants

Reference Number	Study (*n* = 13)	Clear, explicit and appropriate aim	Clearly described context	Researcher reflexivity demonstrated	Source and volume of data appropriate to objectives	Data generation tools well described and appropriate	Analysis approach well described and appropriate	Consideration of limitations and trustworthiness evident	Claims/ findings adequately supported by data	Key concepts relating to migration/ ethnicity are explicit	Summary assessment
[17]	Bhattacharya, 2011	+	+	–	+	+	+	–	–	+	**
[18]	Chakrabarti, 2010	+	+	–	+	+	+	–	+	+	***
[19]	Escandell and Tapias, 2010	+	+	–	+	–	–	–	+	+	**
[20]	Heikkinen and Lumme-Sandt, 2013	+	+	–	–	+	+	–	–	–	**
[21]	Krause, 2008	+	+	–	+	–	–	+	+	+	**
[22]	McFadden, Atkin and Renfrew, 2014	+	+	+	+	–	+	–	+	+	***
[23]	Menjivar, 2002	+	+	–	+	–	–	+	+	+	**
[24]	Sanon, Spigner and McCullagh, 2016	+	+	+	+	+	+	+	+	+	***
[25]	Thomas, 2010	+	+	+	+	+	+	–	+	–	***
[26]	Tiilikainen, 2011	+	+	–	+	+	–	–	+	–	**
[27]	Viruell-Fuentes, 2006	+	+	–	+	–	+	–	+	+	**
[28]	Viruell-Fuentes and Schulz, 2009	+	+	–	+	+	+	+	+	+	***
[29]	Yearwood, 2007	+	+	–	+	+	+	+	+	–	***

Assessment: + (sufficient) and – (insufficient)

Summary assessment: Low (< 4 components assessed as sufficient), Moderate (4–6 components assessed as sufficient), and Good (7–9 components assessed as sufficient)

**Table 3:** Quality assessment – Quantitative or mixed-methods tool - Migrants

Reference Number	Study (*n* = 5)	Clear, explicit and appropriate aim	Clearly described context	Sampling approach appropriate and non-biased	Sample size adequate	Data generation tools well described and appropriate	Analysis approach well described and appropriate	Findings adequately supported by data (not over-stated)	Consideration of limitations, bias and generalizability evident	Key concepts relating to migration/ ethnicity are explicit	Summary assessment
[30]	Baquero, 2010	+	+	–	+	+	+	+	+	–	***
[31]	Hanley, Gravel, Lippel and Koo, 2014	+	–	–	–	–	–	–	–	–	*
[32]	May, 1992	+	+	–	–	+	+	+	+	–	**
[33]	Murphy and Mahalingam, 2004	–	+	+	–	+	–	+	+	–	**
[34]	Plaza and Plaza, 2019	+	+	+	–	–	–	+	+	+	**

Assessment: + (sufficient) and – (insufficient)

Summary assessment: *Low (< 4 components assessed as sufficient), **Moderate (4–6 components assessed as sufficient), and ***Good (7–9 components assessed as sufficient)

**Table 4:** Quality assessment – Qualitative tool – Those who remain behind

Reference Number	Study (*n* = 8)	Clear, explicit and appropriate aim	Clearly described context	Researcher reflexivity demonstrated	Source and volume of data appropriate to objectives	Data generation tools well described and appropriate	Analysis approach well described and appropriate	Consideration of limitations and trustworthiness evident	Claims/ findings adequately supported by data	Key concepts relating to migration/ ethnicity are explicit	Summary assessment
[35]	Amin and Ingman, 2014	+	+	+	+	+	+	+	+	–	***
[36]	Chinouya, 2006	+	+	–	+	+	+	–	–	–	**
[8]	Levitt and Lamba-Nieves, 2011	+	+	–	+	–	–	–	+	+	**
[37]	Mekonnen and Lohnert, 2018	+	+	–	–	–	–	–	–	+	*
[38]	Patzer, 2018	+	+	–	–	+	–	–	+	+	**
[39]	Rubyan-Ling, 2019	+	+	–	–	+	+	–	–	+	**
[5]	Sobiech, 2019	+	+	–	–	+	+	+	+	+	***
[40]	Sriram, George, Baru and Bennett, 2018	+	+	+	+	+	+	+	+	–	***

Assessment: + (sufficient) and – (insufficient)

Summary assessment: *Low (< 4 components assessed as sufficient), **Moderate (4–6 components assessed as sufficient), and ***Good (7–9 components assessed as sufficient)

**Table 5:** Quality assessment – Quantitative or mixed-methods tool – Those who remain behind

Reference Number	Study (*n* = 10)	Clear, explicit and appropriate aim	Clearly described context	Sampling approach appropriate and non-biased	Sample size adequate	Data generation tools well described and appropriate	Analysis approach well described and appropriate	Findings adequately supported by data (not over-stated)	Consideration of limitations, bias and generalizability evident	Key concepts relating to migration/ ethnicity are explicit	Summary assessment
[41]	Battaglia, 2015	+	+	–	+	+	+	+	+	+	***
[42]	Beine, Docquier and Schiff, 2013	+	+	+	+	–	–	–	+	–	**
[43]	Creighton, Goldman, Teruel and Rubalcava, 2011	+	+	+	+	+	+	+	+	+	***
[44]	De, 2013	+	+	–	+	+	+	+	+	+	***
[45]	Diabate and Mesplé-Somps, 2019	+	+	–	+	+	+	–	+	+	***
[46]	Fargues, 2011	+	+	–	–	–	–	–	+	–	*
[47]	Frank, 2005	+	+	+	+	+	+	–	+	+	***
[48]	Lindstrom and Muñoz-Franco, 2005	+	+	–	+	+	+	–	+	+	***
[49]	Lindstrom and Muñoz-Franco, 2006	+	+	+	+	+	+	–	–	+	***
[50]	Roosen and Siegel, 2018	+	+	–	+	+	+	–	+	+	***

Assessment: + (sufficient) and – (insufficient)

Summary assessment: *Low (< 4 components assessed as sufficient), **Moderate (4–6 components assessed as sufficient), and ***Good (7–9 components assessed as sufficient)

### Extraction and synthesis

An extraction template was developed iteratively, piloted and refined in EPPI-Reviewer. The following information was extracted from the articles: general characteristics of the respondents, study design, country where the research was conducted, quality assessment, health outcomes, components of transnational social exchanges and transnationalism/ transnational networks, the link between transnational social exchanges and health. A narrative synthesis was conducted based on the extracted data, defining recurring themes to answer our research questions. A narrative synthesis was adopted suitable to accommodate findings from the diverse types of study (qualitative, quantitative, and mixed-methods). A systematic narrative review will provide a complete, analytical, and objective analysis of the available knowledge and be able to put these outcomes into context [51].

## Results

### Study characteristics

**Fig. 1:**
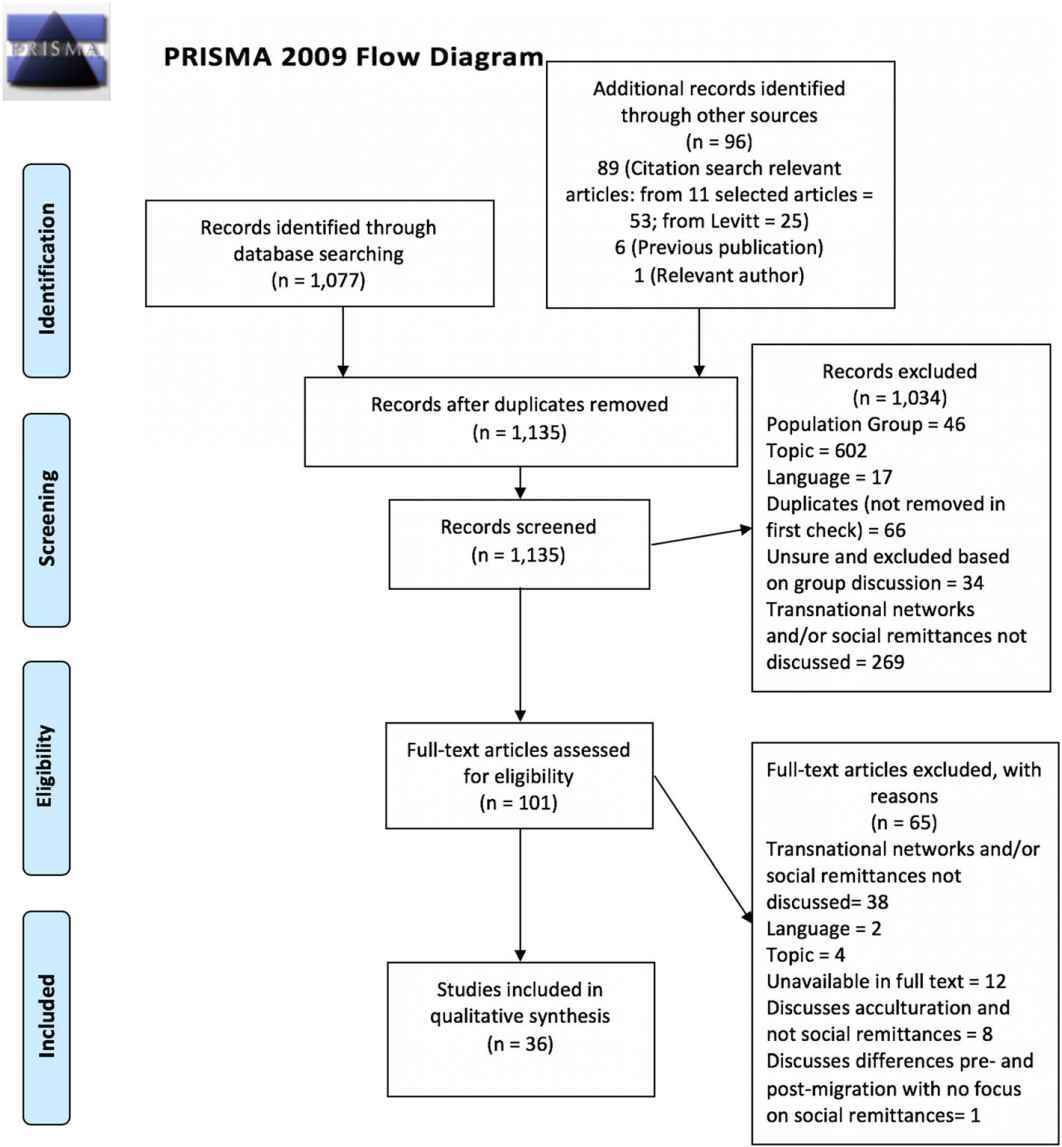
Prisma flow diagram

Figure 1 presents the PRISMA flow chart. From 1173 potential items 1072 were excluded.

Half of the 36 included articles focused on migrant populations (*n* = 18) and the other half focused on those who remain behind (*n* = 18). Thirty-three of the papers were published in a peer-reviewed journal, two were PhD dissertations [5, 30], and one was an international report [45]. The top three health topics that were studied were related to health-seeking strategies (*n* = 9) [18, 19, 23–26, 29, 31, 32], issues related to sexual and reproductive health (*n* = 9) [36, 41, 42, 44–46, 48–50], and healthcare support (*n* = 7) [18, 22, 27, 28, 34, 35, 38].

#### Migrants

The studies on migrants were all conducted in the global North, mainly focusing on the US (*n* = 10) [17, 18, 23, 24, 27–30, 32, 33], but also on Europe (*n* = 6) [19–22, 25, 26], and Canada (*n* = 2) [31, 34]. Thirteen papers adopted a qualitative methodology, predominantly using interviews (*n* = 13) [17–24, 26–28, 32, 34]. The assessed quality of the qualitative articles was moderate (*n* = 7) [17, 19–21, 23, 26, 27] to good (*n* = 6) [18, 22, 24, 25, 28, 29]. The quality of the three quantitative studies was assessed to be mainly of moderate quality (*n* = 3) [32–34]. Two studies used mixed-methods, one was assessed to be of moderate quality [32] and the other of low quality [31] (please see Table 6 for more information).

**Table 6:** Characteristics of included studies - Migrants

Reference number	Study (*n* = 18)	Country	Perspective	# of participants	Data collection	Analysis data	Health focus of study
[30]	Baquero, 2010	US	Mexican migrants	397 households (one respondent per household)	Secondary cross-sectional data from San Diego Prevention Research Center (SDPRC)	Multilevel (hierarchical) logistic regression model	Obesity
[17]	Bhattacharya, 2011	US	Indian migrants	17	Interviews (semi-structured)	Context-driven methodology Axial coding procedure	Acculturative stress
[18]	Chakrabarti, 2010	US	Bengali migrants	40	In-depth, semi-structured interviews	Grounded theory approach	Pregnancy care
[19]	Escandell and Tapias, 2010	Spain	Bolivian migrants	28 transnational families, comprising of 58 individuals	Participant observation and in-depth interviews	Constructivist approach within a transnational framework	Transnational healing strategies
[31]	Hanley, Gravel, Lippel and Koo, 2014	Canada	Precarious status workers (=temporary foreign workers or undocumented migrants)	-Survey: 78 -Interviews: not stated for this study specifically	Surveys and interviews	Not stated	Access to health and health-seeking behaviour
[20]	Heikkinen and Lumme-Sandt, 2013	Finland	Former Soviet Union migrants (Ukraine, Kazakhstan, and Russia)	11	Semi-structured qualitative face-to-face interviews	Content analysis	Transnational connections in later life
[21]	Krause, 2008	UK	Ghanaian migrants	36	-Ethnographic research -Narrative interviews	Not stated	Transnational therapy networks
[32]	May, 1992	US	Middle Eastern migrants (Egypt, Palestine, and Yemen)	73	-Norbeck Social Support Questionnaire - Four Supplementary Social Support Questions - Support System Map -Child Health Interview Guide	- F-tests for independent samples - One-way analysis of variance (ANOVA) - Chi- square - Pearson product moment correlations - Content analysis	Help-seeking for child healthcare
[22]	McFadden, Atkin and Renfrew, 2014	UK	Bangladeshi migrants	-Focus groups: 14 -Interviews: 23	-Focus group discussions -In-depth interviews	Open and inductive coding using an ethnographic approach	Breastfeeding practices
[23]	Menjivar, 2002	US	Ladina and indigenous Guatemalan migrants	26 Also talked to others to complement findings	Participant observation and in-depth, semi-structured interviews	Not stated	Health-seeking behaviour
[33]	Murphy and Mahalingam, 2004	US	West Indian migrants	137	Surveys using the Transnationalism Scale	-Factor analysis -Bi-variate correlations	Mental health
[34]	Plaza and Plaza, 2019	Canada	Trinidadian migrants	-Surveys: 150 (2012) and 100 (2015) -Interviews: 10	-Two online Qualtrics surveys (2012 & 2015) - In-depth interviews	-Interviews: Constant comparative method of analysis -Surveys: Not stated	Transnational care chains
[24]	Sanon, Spigner and McCullagh, 2016	US	Haitian migrants	31	Semi-structured interviews and demographic questionnaire	LeCompte and Schensul’s (2010) approach of qualitative analysis	Hypertension self-management
[25]	Thomas, 2010	UK	Southern African migrants (Zimbabwe, Zambia, and South-Africa)	70	Focus group discussions	Grounded theory approach	Health seeking and treatment behaviours
[26]	Tiilikainen and Koehn, 2011	Finland	Somali migrants	Paper connects 3 different studies: 1) 14 2) 22 main informants 3) Not stated	Paper connects 3 different studies: 1) Interviews 2) Ethnographic research 3) Social field observations and interviews	Paper connects 3 different studies: 1) Not stated 2) Not stated 3) Not stated	Transnational healthcare
[27]	Viruell-Fuentes, 2006	US	Mexican migrants	40	Semi-structured in-depth interviews Participant observations to complement findings	Multistep analytical process	Transnational symbolic and affective characteristics
[28]	Viruell-Fuentes and Schulz, 2009	US	Mexican migrants	40	Semi-structured in-depth interviews	Inductive coding	Transnational social ties and Latino health
[29]	Yearwood, 2007	US	Caribbean migrants	12	Focus groups	Content analysis	Child healthcare decision making

#### Those who remain behind

Articles researching those who remain behind were mainly based in the global South, focusing on North and Central America (*n* = 6) [41, 43, 44, 47–49], Africa (*n* = 5) [5, 36, 37, 39, 45], Asia (*n* = 4) [35, 38, 40, 50], and the Caribbean (*n* = 1) [8]. Furthermore, two of the included articles [42, 46] analysed multiple countries when studying transnational social exchanges and their influences on those who remain behind. Ten out of the eighteen articles [41–50] used quantitative analysis of secondary survey data. Eight of the eighteen articles [5, 8, 35–40] were qualitative. The assessed quality of the qualitative articles was moderate (*n* = 4) [8, 36, 38, 39] to good (*n* = 3) [5, 35, 40]. The quality of quantitative studies was assessed to be mainly of good quality (*n* = 8) [41, 43–45, 47–50]. The included papers for this review focused on the influence of transnational social exchanges on the health of those who remain behind, both on the individual and collective level. Five articles [5, 8, 37, 39, 40] researched collective transnational social exchanges via Diaspora communities or organisations, and thirteen articles [35, 36, 38, 41–50] studied the individual level. It is important to mention that twelve [35, 36, 41–50] of these thirteen studies indirectly presented the perspective of those who remain behind. Two papers [35, 36] addressed this question via interviews with migrants, and ten articles [41–50] via analysis of secondary data (e.g., studying the association between having a migrant household member on the health outcomes of those who remain behind). Please see Table 7 for more information.

### Role of transnational social exchanges in health-related practices and outcomes among migrants

In answering the first component of the research question of this review, five main themes could be identified related to the role of transnational social exchanges on the health of migrants: *Therapeutic effect of the continuing social relationships, disrupted social relationships, hybridisation of healthcare, facilitation of connections to healthcare providers, and factors encouraging or undermining transnational social exchange* (please see Table 8 for more information).

**Table 7:** Characteristics of included studies – Those who remain behind

Reference Number	Study (*n* = 18)	Country	Perspective	# of participants	Data collection	Analysis data	Health focus of study
[35]	Amin and Ingman, 2014	Bangladesh	Those who remain behind (respondents were Bangladeshi migrants in the US)	21	Interviews (in-depth semi-structured questionnaires)	Thematic coding strategy	Eldercare practices and experiences
[41]	Battaglia, 2015	Mexico	Those who remain behind	39,133	Secondary cross data from Mexican National Survey of Demographic Dynamics (ENADID—Encuesta Nacional de Dinámica Demográfica)	IV Regression	Teenage fertility
[42]	Beine, Docquier and Schiff, 2013	Analysis of 175 countries	Those who remain behind (worldwide perspective)	175 countries	Secondary data: -Fertility data (WDI) -Bilateral migration stocks (Parsons et al., 2007) - the skilled-to-unskilled ratio of emigration rates from Docquier, Lowell, and Marfouk, 2009 - Data on from the IMF database urbanisation rate (WDI) - the share of Catholics and Muslims in each source country population, and religious dummies; regions are consistent with the World Bank definition.	-OLS regressions -IV regressions -Dynamic specification model	Fertility
[36]	Chinouya, 2006	Different sub-Saharan African countries (Zimbabwe, Uganda, Nigeria, Zambia, Burundi, Somalia, South Africa, Kenya and Malawi)	Those who remain behind (respondents were sub-Saharan migrants in the UK)	60	Interviews	Framework method	HIV status and transnational childcare taking
[43]	Creighton, Goldman, Teruel and Rubalcava, 2011	Mexico	Those who remain behind	3593	Secondary longitudinal data from Mexican Family Life Survey (MxFLS))	Three-Level Random-Intercept Logistic Regression Models	Overweight / Obesity
[44]	De, 2013	Mexico	Those who remain behind	11,907	Secondary data from ENADID (Encuesta Nacional de Dinámica Demográfica or National Survey of Demographic Dynamics) survey	- Single Equation Probit Models -Instrumental Variable Regression	Contraceptive use
[45]	Diabate and Mesplé-Somps, 2019	Mali	Those who remain behind	5138	Secondary data from ENEM-2009 (Enquête Nationale sur l’Excision au Mali)	-OLS regression -Instrumental Variable Regression	Female Genital Mutilation
[46]	Fargues, 2011	Morocco, Turkey, and Egypt	Those who remain behind	MENA countries	Secondary time-series data on birth rates and migrant remittances	-Demographic analysis -Time correlation	Birth rates
[47]	Frank, 2005	Mexico	Those who remain behind	565	Secondary data from a hospital-based postpartum survey that was implemented in eight different hospitals in Western Mexico (HPS 2001)	Multivariate analysis	Infant health
[8]	Levitt and Lamba-Nieves, 2011	Dominican Republic	Those who remain behind (Dominican Republican Diaspora communities in the US)	50	Semi-structured interviews and 20 years of fieldwork	Not stated	Home-Town Associations (HTA) involvement in health-related projects at COO
[48]	Lindstrom and Muñoz-Franco, 2005	Guatemala	Those who remain behind	2531	Secondary data from 1995 Guatemalan Survey of Family Health (EGSF)	- Multilevel linear regression - Multilevel logistic regression	Contraceptive knowledge and use
[49]	Lindstrom and Muñoz-Franco, 2006	Guatemala	Those who remain behind	1838	Secondary data from 1995 Guatemalan Survey of Family Health (EGSF)	Multi-level logistic regression	Maternal health services utilization
[37]	Mekonnen and Lohnert, 2018	Ethiopia	Those who remain behind (Ethiopian Diaspora communities in Germany)	2 Diaspora associations located in Frankfurt	Key informant interviews, observations, and literature reviews on migration and development	Not stated	Diaspora engagement in health-related development
[38]	Patzer, 2018	-US -Philippines	Those who remain behind (respondents in US and Philippines)	Paper focuses on one case study of a migrant family (US-Philippines), supplemented with other observations	Multi-sited ethnography: participant observation, interviews, and the analysis of the use of new media for migrants in the US and those who remain behind in the Philippines	Not stated	Long-distance care and Food consumption
[50]	Roosen and Siegel, 2018	Afghanistan	Those who remain behind	25,419	Secondary data from cross-sectional data from the Afghan Mortality Survey (2010)	-Ordinary least squares regression -Propensity score matching -IV regression	Birth control knowledge and use
[39]	Rubyan-Ling, 2019	Sierra Leon	Those who remain behind (Sierra Leonean Diaspora communities in the UK)	Interviews: 10	Participant observation, semi-structured interviews	Not stated	Diaspora mobilization during the Ebola outbreak
[5]	Sobiech, 2019	Ghana	Those who remain behind (Ghanaian Diaspora communities in Germany)	Interviews: 50	Semi-structured interviews, observations, and documents	Thematic analysis	Diaspora engagement in health-related development
[40]	Sriram, George, Baru and Bennett, 2018	India	Those who remain behind (different domestic, diasporic, and foreign organisations (e.g., from U.S., U.K., Australia, Singapore, and Saudi Arabia))	-Interviews: 87 -Document review: 248 -Participant observation: 6	In-depth interviews, document review, and non-participant observation of conferences and meetings	Framework method	Transfer of biomedical knowledge

#### Therapeutic effect of the continuing social relationships

Twelve of the eighteen articles, of which four were assessed to be of good [18, 24, 28, 30] and eight of moderate quality [17, 19, 20, 26, 27, 32–34], reported the continuity of social relationships between migrants and their transnational network members, despite the physical distance between them. Continuity of these transnational relationships gave migrants a sense of belonging and provided emotional support.

Continuation of transnational social networks was observed to especially provide emotional care and support to migrants [17, 18, 24, 26–28, 34]. Transnational support, such as providing a sense of community and belonging, was important, especially when migrants experienced isolation, loneliness, marginalisation, cultural mourning, and acculturative stress [17, 20, 27, 28, 34].

*“When you’re homesick it helps you feel connected, sometimes as though you’re still there . . . Pictures posted, FaceTime and chat on Facebook are the most important features for me to show my love.” (Paula – Indo-Trinidadian migrant in Canada)* (34: p.14)

Additionally, three qualitative studies of which two researched Mexican migrants [27, 28] and one studied Bengali migrants [18] in the US, referred to the substantial emotional value of these transnational networks, which played a central role in their daily life, especially for first-generation migrants.

*“Look, our communication, I feel, is not only through the phone, (or) through letters, it is from heart to heart.” (Victoria – Mexican migrant in the US)* (27: p.343)

The terminology used by a few researchers also reflected the value of these transnational networks, symbolically calling them ‘*transnational social therapeutic networks*’ (18: p. 364) and ‘*circuits of affection*’ (27: p.343). Some of the migrant respondents in the good quality qualitative study by Chakrabarti [18] were able to powerfully describe the emotional value and symbolism of their connection with transnational network members and its influence on their wellbeing.

*“So phone card has become like medicine you know.” (Tuhina – Bengali migrant in the US)* (18: p.367)

#### Disrupted social relationships

Five qualitative papers [19, 21, 22, 27, 28] and one mixed-method study [32] reported findings that described alterations to the social relationships between migrants and their transnational network members that were perceived as making them less supportive and useful in case healthcare or support was needed.

Two qualitative studies [19, 27], which were both assessed to be of moderate quality, observed a high secrecy level between migrants and their transnational network members (operating in both directions). The study by Escandell and Tapias [19] revealed the secrecy between transnational network members when migrants would interrupt their interviews with requests such as, *“‘Don’t tell my mother’, ‘Don’t say anything to my sister’, …*” (19: p.413). Migrants preferred to carefully select the information to share with those who remain behind, thereby not always disclosing the truth about their situation. This secrecy came from migrants’ fear that those who remain behind would worry or force them to return [19, 27]. Viruell-Fuentes [27] referred in her research to Renata, a Mexican migrant living in the US, who reported avoiding asking her family in Mexico for support in case of need. Renata believed she was *“*living a better life*”* in the US, which (according to her) precluded her from asking any favours from those who remain behind, as they were living in a less fortunate position [27].

One qualitative paper of good quality [22] and a mixed-methods study of moderate quality [32] observed that migrants missed direct available support and advice from their family members at home. They would especially miss their female kin’s support and advice when raising children and continuing traditional practices (i.e., breastfeeding) [22, 32].

#### Hybridisation of healthcare

Eleven of the eighteen articles [18, 19, 21–26, 29, 31, 32] presented data that described how migrants access and combine diverse healthcare practices, consultations, and advice from destination and origin. The qualitative study of moderate quality by Escandell and Tapias [19] provided a good example of this hybridisation in Alejandro’s case, a Bolivian migrant in Spain who suffered from a herniated disc.

*"Alejandro faxed copies of his MRI scans, x-rays and lab reports to Bolivia. Luís (Alejandro’s brother-in-law, who is a health professional in Bolivia) confirmed the diagnosis and urged him to return to Bolivia to have surgery. He decided to remain on the list, so Luís prescribed some stronger painkillers which Alejandro was able to obtain in Spain on the black market." (Alejandro – Bolivian migrant in Spain)* (19: p.417)

Two qualitative papers of good quality [18, 22], and one mixed-methods paper of moderate quality [32], described how mothers and female family members in the country of origin would provide support. Examples of transnational support were related to pregnancy and childcare. Mothers or other female kin at origin would also pass on health traditions, such as breastfeeding or how to live a healthy pregnancy [18, 22, 32].

*“...yes, I used to call up home regularly... my mother used to tell me some of the things that happen and are expected during pregnancy, comforting me if I got tensed about something...” (Mrinmoyi – Bengali migrant in the US)* (18: p.367)

McFadden et al. [22] described the process by which a Bangladeshi migrant mother, raised in the UK, evaluated the health-related information she received from transnational network members. The mother explained that she did not intend to listen to all the advice (related to breastfeeding) provided by her female relatives. She would evaluate the received information from origin with the retrieved information at destination and see what solution would serve her health and situation best [22].

Different studies that discussed the hybridisation of healthcare indicated diverse moments when migrants would request health-related advice from origin. Nine of the eleven studies that addressed the hybridisation of healthcare, of which four qualitative studies of good quality [25, 30, 32, 36], four of moderate quality [24, 31, 33, 45], and one mixed-methods research of poor quality [18], suggested that migrant respondents would consult their resources from destination and origin simultaneously. The study by Menjivar [23] also highlighted migrants asking for advice from their transnational network members before contacting a health professional at destination. May [32] and Yearwood [29], who both researched child healthcare of migrants in the US, suggested the perceived seriousness of the illness as an important factor prompting migrants to search for either professional help at destination or help within their (transnational) social networks. If migrant parents consider a disease to be too serious, they would immediately reach out to the healthcare professional at destination; however, if the illness were found to be none-life-threatening, they would first contact their (transnational) network members before searching for further professional help in their place of destination [29, 32].

#### Facilitation of connections to healthcare providers

Five qualitative studies from which one was assessed as good quality [25] and four as moderate [19, 21, 23, 26], suggested the importance of family members’ input in migrants’ healthcare decisions. Thomas [25] studied the health-seeking and treatment behaviour of South African migrants in the UK. Nina, one of the focus-group members in the study, reflected on the crucial role of family members in health decision making. She described why family members back home should be contacted when a migrant in the UK needs to go to a hospital, especially when experiencing mental illness. It would be up to the family members’ advice in South Africa to recommend whether or not the migrant needed to return for healthcare and the following steps they would need to take to recover [25].

Four qualitative articles studying transnational healing and healthcare practices of migrants in Europe [19, 21, 25, 26] and one qualitative paper researching the health-seeking behaviour of Guatemalan migrants in the US [23] discussed the role of transnational network members facilitating connections between the migrant and healthcare providers in their country of origin. The studies suggested the essential role of transnational network members in connecting migrants with traditional healers. Migrants could attend the recommended healing sessions by returning to their home country. When migrants could not return, the consultation and healing sessions with the traditional healers would take place “transnationally”, either via telephone or internet or via the transnational network members who would visit the traditional healer in their name. These remote healing sessions were believed to work because spiritual forces would not be limited by physical borders [23, 26]. Studies [19, 21, 25, 26] reported that migrants generally experienced positive results from traditional healing practices. Lucia, a Bolivian migrant in Spain in the qualitative study of moderate quality by Escandell and Tapias [19], described a moment when she had continuous headaches, which alarmed her family members at home. Without informing her, her parents consulted a traditional healer in Bolivia, who conducted a diagnostic ritual on a piece of Lucia’s clothing. Based on this diagnosis, Lucia received instructions from her transnational network members to receive transnational healing. After this, her headaches vanished, which she attributed to the transnational healing [19].

Migrants would also return to receive professional healthcare at origin, as was discussed in the qualitative study of moderate quality by Menjivar [23]. Rosa, a Guatemalan migrant in the US, suffered from abdominal pain. She contacted a health professional in the US who prescribed medication. Her pain, however, would not diminish after taking the prescribed medicine. In agreement with her mother and sister in Guatemala, Rosa decided to return and was operated on her gall bladder, which finally stopped her pain [23].

#### Factors encouraging or undermining health-related transnational social exchanges

Thirteen articles [18, 19, 21–23, 25–29, 31, 32, 34] suggested encouraging or undermining factors linked to health-related transnational social exchanges.

#### Encouraging factors

Factors that would encourage health-related transnational social exchanges could be related to the perceived structural barriers accessing healthcare at destination and migrant status [18, 19, 22, 23, 25–29, 31, 32, 34].

##### Perceived structural barriers accessing healthcare at destination

Five studies [19, 23, 25, 26, 31] described the perceived structural barriers to accessing healthcare for migrants within their destination country. Important to note the variation in quality in these studies, where the qualitative studies were assessed as moderate [19, 23, 26] to good quality [25], the mixed-methods study [31] was assessed to be of poor quality.

Migrants would perceive their transnational resources as trustworthy and approachable in searching out health-related advice, information, and care [19, 23, 31]. In the study by Menjivar [23], a Guatemalan migrant who returned home for an operation explained that the reasons for her return were based on low-quality care, her mistrust of the healthcare, and the experienced misunderstanding and discrimination while accessing healthcare in the US.

*“Just because we are poor and don’t understand the language, doctors here think that we don’t have a right to be cured. So they tell you that you have one little thing when in fact you may die.” (Rosa – Guatemalan migrants in the US)* (23: p.457)

In studying the relevant articles for the narrative review, a few qualitative papers [25, 26] described spirituality and religiosity as important components for migrant health and wellbeing. In their search for health, migrants might feel misdiagnosed, or they might even feel the proposed cure at destination to be improper for their illness (especially for mental health), as they relate certain diseases to these other components [25, 26].

*"In our culture, almost every mental health problem is related to witchcraft or spirits, or demons or probably that it’s like a curse. If you go the hospital it’s not going to go away, you have to go to a healer." (Donald – Zimbabwean migrant in the UK)* (25: p.610)

##### Migrant status

Two qualitative papers of moderate quality [19, 21] and one mixed-methods paper of low quality [31] reflected on the role of migrant status in shaping access to healthcare at destination. The study by Hanley et al. [31] researched precarious status workers, who mentioned their status would hinder them from accessing healthcare in Canada and their high dependency on their employer in accessing healthcare. When being ill, migrants would fear their contract would not be renewed, meaning a loss of income that they and their family who remain home depend on [31].

*“What happens is that the company doesn’t take you to the hospital when you want, but when they want to... They took me to the hospital and all they did was give me some medicine so that I could keep working.” (Interview S07) (Migrant worker in Canada)* (31: p.11)

#### Undermining factors

Four qualitative studies of moderate [19, 27] and good quality [22, 25] and one mixed-methods study of moderate quality [32] indicated relevant factors undermining health-related transnational social exchanges, including changes in healthcare beliefs and scepticism of traditional practices [19, 22, 25, 27, 32].

Thomas [25] described varied findings across UK-based South African migrant respondents. Notwithstanding the importance of traditional healing practices reported by some, others expressed scepticism about these practices, which they believed did not conform to their current religious beliefs. A few respondents also complained about the lack of authenticity and cleanliness of those performing the transnational traditional healthcare practices [25]. These changes in attitudes towards traditional healthcare approaches meant that some migrants were not inclined to request or accept traditional healthcare advice from transnational network members.

Two qualitative studies [19, 22] described migrants’ changing perceptions of traditional health practices. The study by McFadden et al. [22] described the grandmothers’ reflections of young Bangladeshi migrant mothers residing in the UK and their resistance to (transnational) female family members’ breastfeeding-related advice.

*“G9 (Grandmother of Bangladeshi migrant in the UK): Whatever was practiced in the olden days has changed like everything previously was followed by listening to the elders and nowadays the daughters-in-law don't do that. They would not listen to the in-laws.”* (22: p.444)

### Role of transnational social exchanges in shaping health-related practices and outcomes among those who remain behind

Four main themes could be identified in relation to the role of transnational social exchanges in the health of those who remain behind: *Transnational transfer of health-related advice, norms, and support; associations between ‘migrant exposure’ (being part of a migrant household) and health behaviours/outcomes; transnational collective transfer of health knowledge*; *and power and resistance in health-related transnational social exchanges* (please see Table 9 for more information).

**Table 8:** Results – Migrants

Reference Number	Study (*n* = 18)	Therapeutic effect of the continuing social relationships (12/18)	Disrupted social relationships (6/18)	Hybridisation of healthcare (11/18)	Facilitation of connections to healthcare providers (5/18)	Factors encouraging or undermining health-related transnational social exchanges (13/18)
[30]	Baquero, 2010	x				
[17]	Bhattacharya, 2011	x				
[18]	Chakrabarti, 2010	x		x		x
[19]	Escandell and Tapias, 2010	x	x	x	x	x
[31]	Hanley, Gravel, Lippel and Koo, 2014			x		x
[20]	Heikkinen and Lumme-Sandt, 2013	x				
[21]	Krause, 2008		x	x	x	x
[32]	May, 1992	x	x	x		x
[22]	McFadden, Atkin and Renfrew, 2014		x	x		x
[23]	Menjivar, 2002			x	x	x
[33]	Murphy and Mahalingam, 2004	x				
[34]	Plaza and Plaza, 2019	x				x
[24]	Sanon, Spigner and McCullagh, 2016	x		x		
[25]	Thomas, 2010			x	x	x
[26]	Tiilikainen, 2011	x		x	x	x
[27]	Viruell-Fuentes, 2006	x	x			x
[28]	Viruell-Fuentes and Schulz, 2009	x	x			x
[29]	Yearwood, 2007			x		x

**Table 9:** Results - Those who remain behind

Reference Number	Study (*n* = 18)	Transnational transfer of health-related advice, norms, and support (4/18)	Associations between 'migrant exposure'(being part of a migrant household) and health behaviours/ outcomes (8/18)	Transnational collective transfer of health knowledge (5/18)	Power and resistance related to health-related transnational social exchanges (6/18)
[35]	Amin and Ingman, 2014	x			x
[41]	Battaglia, 2015		x		
[42]	Beine, Docquier and Schiff, 2013	x			
[36]	Chinouya, 2006				x
[43]	Creighton, Goldman, Teruel and Rubalcava, 2011		x		
[44]	De, 2013		x		
[45]	Diabate and Mesplé-Somps, 2019		x		
[46]	Fargues, 2011	x			
[47]	Frank, 2005		x		
[8]	Levitt and Lamba-Nieves, 2011			x	
[48]	Lindstrom and Muñoz-Franco, 2005		x		
[49]	Lindstrom and Muñoz-Franco, 2006		x		
[37]	Mekonnen and Lohnert, 2018			x	
[38]	Patzer, 2018	x			x
[50]	Roosen and Siegel, 2018		x		
[39]	Rubyan-Ling, 2019			x	x
[5]	Sobiech, 2019			x	x
[40]	Sriram, George, Baru and Bennett, 2018			x	x

#### Transnational transfer of health-related advice, norms, and support

Two qualitative studies of moderate [38] and good quality [35], and two quantitative studies of moderate [42] and low quality [46] indicated the transnational transfer of health-related advice, norms, and support from migrants to those who remain behind.

The two qualitative studies [35, 38] referred to health-related advice or sometimes even instructions and emotional support. The multi-sited ethnographic research by Patzer [38] helped to clarify the long-distance care between Philippine migrants in the US and their kin who remain behind through balikbayan boxes and related transnational dietary advice. Migrants would consciously provide clear instructions on how to use their economic remittances for food.

*“She (Auring – Philippine migrant in the US) is speaking with a clear American accent. The girl in front of the computer is her niece, Nicole. The conversation (via Skype) follows:*

*– Please tell your mother to give the $ 200 to Mr. Ed and get the rice.”* (38: p.142)

The qualitative research by Amin and Ingman [35] studied the care practices of Bangladeshi migrants in the US towards their elderly parents residing in Bangladesh. Here, migrant children were found to especially provide healthcare advice and emotional support to their parents.

*“I call my parents 2–3 times a week. I enquire about their health. My father has diabetes and a heart condition. I always remind him to take medicine on time, check the sugar, walk, and eat appropriately.” (Bangladeshi migrant residing in the US)* (35: p.319)

The quantitative studies by Beine et al. [42] and Fargues [46] analysed secondary data on a large-scale examining the relationship between international migration and fertility and birth rates. Fargues [46] analysed secondary time-series data from Morocco, Turkey, and Egypt, finding a strong correlation between migrant remittances and birth rates in the country of origin. These changes in birth rates would depend on the existing norms of the country of destination, with increasing birth rates at origin related to migration to the Gulf and decreasing birth rates at origin with migration to the global North [46]. Beine et al. [42] constructed a dataset based on secondary data of 175 countries, observing a significant association between international migration and fertility rates. Similar to Fargues [46], Beine et al. [42] inferred these outcomes were a result of the diffusion of fertility norms from migrants to their origin countries, suggesting that the context where migrants live and come from was a relevant factor in the transfer of fertility norms.

*“Our main finding (based on an Ordinary Least Squares regression analysis and an Instrumental Variables regression analysis) is that international migration results in a transfer of fertility norms from host to migrants' home countries, resulting in a decrease (increase) in home country fertility rates if they are higher (lower) than host country rates.”* (42: p.1407)

#### Associations between ‘migrant exposure’ and health behaviours and outcomes

Eight quantitative studies of good quality [41, 43–45, 47–50] applied the analysis of secondary survey data to explore the possible influence of social remittances on the health of those who remain behind. When using secondary data, direct measures of the receipt of social remittances were not available, so that proxies were used, mainly ‘having a migrant household member’. These studies reported a variety of associations.

The study by Battaglia [41] analysed secondary cross-sectional data, identified that living in a migrant household reduced the likelihood of teenage pregnancies and increased the likelihood of child delivery by a professional healthcare worker and contraceptive knowledge by those who remain behind in Mexico. Creighton et al. [43] analysed secondary longitudinal data where they observed a higher likelihood of becoming obese or overweight when Mexican children had a migrant household member living in the US. Using secondary cross-sectional data, the study by De [44] identified positive associations between being part of a migrant household in Mexico and the use of modern birth control. While, Frank [47], using secondary cross-sectional data, described positive associations between Mexican women with migrant spouses and pregnancy practices, including receiving prenatal care, exercising, and taking multi-vitamins. Frank [47] also identified that Mexican women who had a migrant husband were less likely to smoke and were less likely to breastfeed than those who did not have a migrant husband. Lindstrom and Muñoz-Franco performed two studies on the link between transnational social exchanges and the health of women who remain behind, using secondary cross-sectional data [48, 49]. In their study of 2005, Lindstrom and Muñoz-Franco observed a positive association between living in a migrant household and modern contraceptive knowledge and use in Guatemala [48]. Their study of 2006 analysed the association between having a migrant household member and the use of formal prenatal care and delivery assistance, finding positive associations for both outcomes [49].

Two quantitative studies [45, 50] identified both health-promoting and health-harming associations, suggesting the importance of the country context for migrants and those who remain behind, such as healthcare access and ruling norms when interpreting the results. Diabate and Mesplé-Somps [45] observed that return migration reduced the likelihood of female genital mutilation in Mali. They found this result was mainly driven by migrants who returned from Côte d’Ivoire. In the study by Roosen and Siegel [50], different associations were observed depending on cultural background and destination countries. Migration to Iran increased the likelihood of birth control knowledge of non-Pashtun women who remain behind in Afghanistan. While migration to Pakistan reduced the likelihood of birth control knowledge and use of Pashtun women who remain behind in Afghanistan [50].

#### Transnational collective transfer of health knowledge

Transfer of healthcare knowledge was observed not to be solely on the individual level, but five qualitative articles of good [5, 40], moderate [8, 39], and low quality [37] also identified this on a collective level from diaspora members to those who remain behind. Diasporic groups were found to be active in supporting healthcare projects in their country of origin. Diverse health-related activities from the diaspora were observed, such as promoting exercise, providing health education about taboo topics in health fairs, transferring experiences between health experts, providing advice and support during the Ebola outbreak, training healthcare students, and establishing emergency medicine [5, 8, 37, 39, 40]. Migrants especially showed commitment to improving healthcare provision in their countries of origin. As an example, the qualitative study by Rubyan-Ling [39] documented the engagement at different levels of several Sierra Leonean diaspora members residing in the UK during the Ebola outbreak.

*“I basically wrote a few passages and came up with a small piece, which I put on Facebook, which I circulated with family and friends.” (Charles – Sierra Leonean migrant in the UK)* (39: p.224)

*“I started calling everyone I was in touch with, advising them what to do, what not to do.” (Ernest – Sierra Leonean migrant in the UK)* (39: p.227)

#### Power and resistance in health-related transnational social exchanges

Six qualitative studies of good [5, 35, 40] and moderate quality [36, 38, 39] identified power and resistance in health-related transnational social exchanges.

Patzer [38] observed the use of information technologies by migrants to control those who remain behind. With sending remittances and providing clear health-related instructions, migrants feel the need to check the adherence to their instructions from those who remain behind. This constant control could result in avoidance-behaviour of those who remain behind, finding excuses to not communicate (with a camera) with the migrant, avoiding the camera, or not even responding to the migrant when being in a Skype call [38]. Patzer [38] also stressed the relevance of migrant type on perception and acceptance at home. In the Philippines, they identify two types of migrants, one is the Balikbayan who already has been gone for a long time and is well-established in his or her new country, and the other is the Overseas Contract Worker, who is a hard-working temporary migrant suffering during his or her time abroad. Remittances of any kind were received differently from these two types of migrants - being accepted more positively from Overseas Contract Workers, while being resisted or resented from Balikbayans’ who were even perceived as neo-colonisers [38].

Not all health-related transnational engagement was perceived as positive by those who remain behind [5, 39, 40]. The study by Sobiech [5] observed a mismatch of expectations between Ghanaian migrants in Germany and those who remain behind in organising transnational healthcare projects in Ghana. Conflicting desires around improvements to healthcare and general expectations of each other lead to friction between the two groups [5]. During the Ebola outbreak, the respondents of the study by Rubyan-Ling [39] described actions by some diaspora members who engaged in circulating rumours on Facebook about Ebola, negatively influencing the efforts of other diaspora members [39]. Sriram, George, Baru, and Bennett [40] also observed tensions between those who remain behind and diaspora members due to power imbalances between nationals and internationals, leading to a reduced role of diaspora members in their development efforts.

Two studies [35, 36] referred to the non-disclosure of information. The qualitative study of moderate quality by Chinouya [36] analysed how sub-Saharan African migrants who were HIV-positive would provide information to their children either living with them in the UK or at origin. Chinouya [36] observed that migrants would request caregivers at origin to take their child for an HIV test. Children were not always informed they had had this test, the test results, or even the HIV status of their parents. When the child lived in the country of origin, parents preferred not to disclose this information hoping to protect the child from possible negative consequences and wanting to discuss in person something as delicate as their HIV-status [36].

*‘I cannot tell her on the phone, she has to see my face and show her my emotions how I feel about it. I feel I should be there. Face to face.’ (mother)* (36: p.14)

## Discussion

This study investigated the role of transnational social exchanges in shaping health-related practices and health outcomes among migrants and those who remain behind, using a systematic narrative literature review approach.

### Role of transnational social exchanges in health-related practices and outcomes among migrants

Transnational social relationships of migrants were observed to be continued despite physical distance as well as being disrupted, both influencing the emotional wellbeing of migrants. When living in a foreign country, migrants frequently experience isolation, loneliness, marginalisation, cultural mourning, and acculturative stress. Mainly these mental health stressors are why migrants experienced transnational care and support as especially valuable in maintaining a sense of belonging. These observations could reflect the increased stress and anxiety related to the acculturation process. Research has observed that migrants who identified themselves more with the host society showed increasingly more health literacy and health benefits. Although these higher levels of acculturation could also be related to several undesirable health behaviours copied from the host society [52].

While finding their way within a new society, migrants continuously find themselves balancing their identity between their host and home country’s cultures [53]. Migrants would evaluate the received healthcare advice at destination and origin to fit their situation best, resulting in hybrid health-seeking strategies and adaptations in their health behaviours. Transnational family members were observed to also play an essential role in healthcare decision-making and connecting migrants to healthcare providers or healers at origin.

Reasons why migrants used transnational networks and traditional healers appeared mainly based on obstacles when accessing healthcare at destination and migrant status. Therefore, they perceived their transnational networks as more trustworthy and approachable sources in their search for health. Similar challenges (e.g., cultural and communication barriers) were identified for other minority groups, such as Indigenous and low SES populations. These overlapping barriers reveal the role of social marginalisation and low health literacy on healthcare access and utilisation [53–55]. Interestingly, in the study of Priebe et al. [56], healthcare professionals identified similar obstacles when providing healthcare to migrant patients.

Several papers reported that migrants perceive spirituality and religiosity as important to their health and wellbeing; elements that are often overlooked in Western biomedical approaches. Similar understandings are also found in Indigenous populations in North America, Australia, and New Zealand, believing that the imbalance of one’s spirit is the origin of illness and seeking traditional health practices [55]. Similarly, it is possible that in their search for health, migrants might miss the additional components to be addressed at destination or they might feel misdiagnosed as they relate certain illnesses to these other components. Possibly they try to fill these gaps by contacting transnational network members or traditional healers. The use of transnational traditional healing practices to deal with their illnesses was especially relevant for mental health. Migrants could receive the traditional healing transnationally, or they would return for a specific healing period. There are some indications that transnational healing is an emerging business in countries of origin [25, 26].

Previous studies [57, 58] did not research the role of transnational social exchanges in health but studied migrants’ health information-seeking behaviour. Both studies observed the role of internet sites and forums in providing health-related information to migrants in their native language. Seo et al. [58] found that migrant women would especially consult these online forums to talk to migrant women who were living in a similar situation as them. It appeared that the migrants considered other migrant women to be more relatable than those who remain behind [58]. Depending on their identity transition, this could mean that migrants would not necessarily consult transnational network members, as they might not relate (anymore) to their counterparts who remain behind – possibly diminishing the role of transnational networks in providing health-related information [52].

Though not identified as one of the main health topics, mental health and wellbeing was often intertwined with other health topics, either directly or indirectly. Also, the terminology used in studies when referring to transnational networks, such as ‘transnational social therapeutic networks’ and ‘circuits of affections’, reflects its emotional weight and importance to migrants and those who remain behind. It was also observed that, especially first-generation migrants, found mental support via transnational networks essential. Depending on the phase of their life, mothers would often provide emotional support to their migrant daughters during and after their pregnancy.

### Role of transnational social exchanges in shaping health-related practices and outcomes among those who remain behind

In the redefinition of social remittances, Levitt and Lamba-Nieves [8] specified the different levels on which social remittances can occur, the individual and collective. These two levels of transnational social exchanges were also identified in our narrative review. Also, as redefined by Levitt and Lamba-Nieves [8], positive and negative observations have been suggested in this review of transnational social exchanges on the health of migrants and those who remain behind. Migrants and diaspora organisations were observed to provide care and support to those who remain at origin, as was also reflected in one of the main health topics of the included studies of this review. Furthermore, health access and existing norms at destination could expose migrants to new or other health behaviours that they might share with their transnational network members, consequently possibly influencing health practices and outcomes of those who remain behind. Although, some studies of this review also observed resistance of those who remain behind to the transnational social exchanges from migrants or diaspora organisations. The possible adoption, rejection, or re-invention of these transnational social exchanges from migrants to those who remain behind could also be related to the stages (trial, enmeshment, and negotiation) of the identity management process from migrants and those who remain behind [52].

Transnational social exchanges potentially shape health-related practices for both the migrants and those who remain behind. Social remittances are considered to happen consciously between transnational network members [8, 9]. This review did observe some conscious transnational social exchanges and some other social exchanges that did not necessarily appear to be conscious, but more a natural exchange between loved ones sharing support and advice during a daily conversation. Therefore, it can be questioned whether all transnational social exchanges can be considered to be conscious.

### Limitations and recommendations

Even though this study provides insight into the role of transnational social exchanges on the health of migrants and those who remain behind, some limitations must be acknowledged. The limitations will be discussed both in relation to our review methodology and in relation to the available body of literature.

At the beginning of our review-process, it became clear that researchers from different disciplines initially had different understandings of the main concepts (social remittances and transnationalism, what to include and exclude related to health). By cooperation and thorough discussions on the topics, any misunderstandings were clarified, and we have been able to provide a strong base for our review. It is therefore recommended that future research on this topic should make use of multi-disciplinary teams. Also, this study did not exclude papers that were assessed to be of low quality, which might limit our implications. However, we addressed the quality of the studies when discussing the results in our review. Also, we acknowledge that by only including English articles, we have limited our results on the existing literature that might be available on this topic. During our literature search, we came across studies researching the migration and transnational social exchanges between the US and Latin American countries, and similar studies on EU migration. Unfortunately, none of the authors speak the languages in which some of these studies are published, such as Spanish, Russian, Polish, Czech, Bulgarian, or Romanian. As some of the studies included in our narrative review analysed the migrants and those who remain behind within the US - Latin American context, we could assume the majority of our findings could cover their results. However, only one study discussed the migrant perception within the EU [20]. The reflection of migration experiences within the EU from migrants from ‘newer’ Member States to ‘older’ Member States are limited in this research. Studying these experiences could be interesting as the EU has open borders, potentially leading to additional findings on this topic.

Also, some limitations relating to the body of literature were apparent, such as the focus of the included studies on women. As a result of this focus, several other perspectives are not presented or underrepresented, such as men, children, elderly, and non-binary individuals. In addition, papers tended to have a narrow focus on a few health issues, restricting the findings to the discussed health areas. Furthermore, it was impossible to assess the relative importance of transnational networks versus local networks as none of the included papers addressed this. There were no quantitative studies that measured the actual transnational social exchanges of information nor its effects on health; all of them inferred to this. Additionally, the included articles mainly used single-site studies, making it difficult to grasp such a complex process as transnational social exchanges. Also, the studies of those who remain behind often could not truly reflect the voice or the reality of those who remain because these studies mainly focused on migrants’ perceptions or used secondary data that suggested the influence of migrant exposure. The final limitation related to the body of literature will stress the clear division of geographic locations between the studies focusing on migrants (global North) and those who remain behind (global South). This division indicates the prioritisation of research output based on South-to-North migration but does not reflect the reality of global migration patterns [59–61]. Migration is diverse in its movements between and within the global North and South; by missing this aspect, we miss a genuine global perspective on transnational social exchanges and their influence on the health and wellbeing of migrants and those who remain behind [59, 61, 62]. Furthermore, separating and associating research output of the global North with receiving developed countries and the global South with those who remain behind in developing countries provides another distorted perspective of the realities and potential of migration and transnational social exchanges [62].

As this was initial research synthesising the current studies on the topic, it was chosen for a narrative review to map all available studies using different methods. By using narrative analysis, the presented outcomes can provide recommendations for future policies and interventions [51]. This review has possible implications for clinicians and policymakers. It is important to understand and acknowledge the transnational life migrants live and its potential influences on health. Furthermore, it is important to understand the use of transnational networks in health advice, consultations, and even (traditional) healing. Also, the prominent role of family members in healthcare decision making should be considered. Healthcare professionals need to recognize and be equipped with a better understanding and tools to respond to traditional medication and healing practices as well as differences in migrant’s perceptions of health and healing. This review also raises issues around shared decision making, especially for minority populations with possibly low health literacy. When countries of destination invest in migrants’ health, there is a possibility to create a domino effect of their investments on the health of those who remain behind. Policies investing in migrants’ health could be specifically related to the main components of the human right to the highest attainable standard of health, namely availability, accessibility, acceptability, and good quality healthcare and healthcare-related information [63]. In reducing health disparities in destination countries, cultural competence skills-building for healthcare providers and migrant patients could be a recommended strategy to build bridges [52].

Extra research on the role of transnational social exchanges on health and wellbeing is highly recommended, including a broader approach to target populations and health areas. Future research on this topic is strongly recommended to consider societies outside the dominating global North to close the North-South research focus gap [64]. Balanced research reflecting the world’s reality and connecting knowledge of both global sides is required when studying transnational social exchanges [60, 62]. This review also suggests a number of specific areas that deserve further investigation, including: the hybridisation of health practices; the influence of identified barriers (accessing healthcare at destination, migrant status, and defining health and healing from a migrant perspective) on transnational social exchanges; the role of destination countries in providing healthcare and health-related information to migrant populations and its possible consequences on the health of those who remain behind via transnational social exchanges; and transnational traditional healing as an emerging business in countries of origin. To get a complete picture of transnational social exchanges and its influences on health, it is recommended that researchers study both perspectives simultaneously, migrants and those who remain behind, by using mixed-methods studies and multi-sited ethnographic approaches. To shed light on the social processes explored it is recommended to engage sociological concepts and theories when researching transnational social exchanges.

## Conclusion

This paper is the first systematic narrative literature review to examine what is known about the roles of transnational social exchanges in the health-related practices and health outcomes of migrants as well as those who remain behind. This research observed that transnational social exchanges potentially could be supportive or disruptive on the health of migrants and those who remain behind. The quality of the majority of the included studies was assessed to be moderate to good, and it has been observed that in the last fifteen years, more attention has been given to the topic. However, the identified differences in geographic locations and methods used when studying the two groups is still a point of attention for future studies, as well as the observed lack of representation of the perspective of those who remain behind. Policy makers and healthcare practitioners should seek to understand and be responsive to transnational social exchanges, given their implications for the health of both migrants and those who remain behind.

## Data Availability

Supporting information is included in the body of the manuscript and in Tables 2, 3, 4, 5, 6, 7, 8 and 9. Additional data used and/or analysed during the current study is available from the corresponding author on reasonable request.
